# The water catalysis at oxygen cathodes of lithium–oxygen cells

**DOI:** 10.1038/ncomms8843

**Published:** 2015-07-24

**Authors:** Fujun Li, Shichao Wu, De Li, Tao Zhang, Ping He, Atsuo Yamada, Haoshen Zhou

**Affiliations:** 1 Energy Interface Technology Group, National Institute of Advanced Industrial Science and Technology (AIST), 1-1-1, Umezono, Tsukuba 305-8568, Japan; 2 Department of Chemical System Engineering, The University of Tokyo, 7-3-1, Hongo, Bunkyo-ku, Tokyo 113-8656, Japan; 3 National Laboratory of Solid State Microstructures & College of Engineering and Applied Sciences, Nanjing University, Nanjing 210093, China

## Abstract

Lithium–oxygen cells have attracted extensive interests due to their high theoretical energy densities. The main challenges are the low round-trip efficiency and cycling instability over long time. However, even in the state-of-the-art lithium–oxygen cells the charge potentials are as high as 3.5 V that are higher by 0.70 V than the discharge potentials. Here we report a reaction mechanism at an oxygen cathode, ruthenium and manganese dioxide nanoparticles supported on carbon black Super P by applying a trace amount of water in electrolytes to catalyse the cathode reactions of lithium–oxygen cells during discharge and charge. This can significantly reduce the charge overpotential to 0.21 V, and results in a small discharge/charge potential gap of 0.32 V and superior cycling stability of 200 cycles. The overall reaction scheme will alleviate side reactions involving carbon and electrolytes, and shed light on the construction of practical, rechargeable lithium–oxygen cells.

## Abstract (web summary)

The main challenges in lithium-oxygen batteries are the low round-trip efficiency and decaying cycle life. Here, the authors present that a trace amount of water in electrolytes facilitates oxygen cathode reactions, enabling the batteries to be operated with small overpotential and good cycling stability.

## Graphical abstract

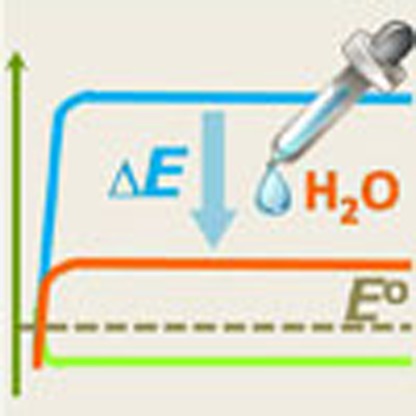

The Li–O_2_ cells are operated with oxygen reduction to produce Li_2_O_2_ and its reverse oxidation to release Li^+^ ions and oxygen at cathodes (2Li^+^+O_2_+2e^−^↔Li_2_O_2_, *E*°=2.96 V vs Li^+^/Li) in discharging and charging processes, respectively^1^,^2^,^3^. The intrinsic chemical and physical nature of Li_2_O_2_ and its intermediates can lead to instability of other cell components, such as carbon supports (>3.50 V)^4^ and electrolytes, and formation of detrimental byproducts, typically Li_2_CO_3_ and lithium alkyl carbonates^4^,^5^,^6^,^7^,^8^,^9^. They can subsequently induce high overpotentials in both discharging and charging processes and eventually discharge/charge failure during cycles^4^,^5^,^6^,^7^,^8^,^9^,^10^,^11^,^12^,^13^. A lot of efforts have been made to address these issues, but there are still many challenges for a practical Li–O_2_ cell^4^,^5^,^6^,^7^,^8^,^9^,^10^,^11^,^12^,^13^,^14^.

The success of deploying Li–O_2_ cells in the future will be crucially dependent on how small the overpotentials at the cathode side can be reduced and how many cycles they can work reversibly^15^,^16^,^17^. Currently, two strategies have been widely applied for these issues. One is to use carbon-free or carbon-alternative cathodes, like nanoporous gold^10^, TiC^11^ and conductive metal oxide supported Ru^12^,^13^, which can circumvent side reactions involving carbon. The charge overpotentials ensuring the decomposition of Li_2_O_2_ are reduced to ∼0.54 V, corresponding to charge potentials of ∼3.50 V (refs 10, 11, 12, 13). This direct electrochemical oxidation of Li_2_O_2_ was revealed to involve multiple processes, in which the initial delithiation occurring at an overpotential of 0.44 V, possibly suggests the theoretical limit^18^,^19^. Alternatively, redox mediators have been introduced to chemically oxidize Li_2_O_2_ and the apparent charge potentials are typically determined by the mediators. For instance, tetrathiafulvalene and LiI reduce the charge potential to ∼3.50 V, equal to a charge overpotential of 0.54 V (refs 20, 21, 22, 23). In principle, Li_2_O_2_ can be oxidized by mediators possessing higher redox potentials than its equilibrium potential; however, practical mediators are very limited. A more efficient and compatible one for further potential reduction is still lacking. By either the direct or indirect (mediators) Li_2_O_2_ oxidation strategy, it will be a great challenge to reduce the charge potentials to feasible values. Exploring new reaction mechanisms at the oxygen cathodes to significantly improve the round-trip efficiency and cycling stability is essential and urgently necessary for the development of Li–O_2_ cells.

Although water in electrolytes has been found to affect the morphologies of discharge products and increase the discharge capacity^24^,^25^,^26^, its presence in electrolytes or O_2_ atmosphere resulted in rapid charge potential increase and hence cell death after several cycles^26^,^27^,^28^. In addition to the main discharge product Li_2_O_2_, a byproduct LiOH was also detected^27^. We found that the decomposition of LiOH is strongly related to the applied catalysts, such as Ru nanoparticles supported on Super P (Ru/SP). To reduce the charge overpotentials of Li–O_2_ cells, a trace amount of water in electrolytes and electrolytic mangnese oxide (EMD, γ-MnO_2_) nanoparticles are both utilized to favour the transformation of the discharge product from Li_2_O_2_ to LiOH and its following decomposition on charging. The MnO_2_ incorporated in Ru/SP (Ru/MnO_2_/SP) also allows for the water regeneration at the cathode during the discharge/charge cycles. This enables the Li–O_2_ cell to operate with a small discharge/charge potential gap and superior cycling stability. A reaction mechanism by converting Li_2_O_2_ to LiOH in the presence of both water and Ru/MnO_2_/SP is proposed.

## Results

### Low overpotentials at Ru/MnO_2_/SP

The Li–O_2_ cells were constructed with Ru/MnO_2_/SP pressed onto a carbon paper as cathodes, 0.5 M LiClO_4_ in DMSO containing 120 p.p.m. of H_2_O as electrolytes and LiFePO_4_ in replacement of Li anodes. The LiFePO_4_ is not a practical anode, but is able to avoid the reaction of Li metal and the trace amount of H_2_O and any contamination from formation of the solid electrolyte interface layer on the Li anode^20^. It has a stable potential of ∼3.45 V vs Li^+^/Li regardless of the state of charge and favours the investigation on the discharge/charge behaviour in the presence of H_2_O and the underlying reaction mechanism specifically at the oxygen cathode. The discharge/charge potentials at the oxygen cathode of the Li–O_2_ cells are converted to against Li for discussion.

**Figure 1:**
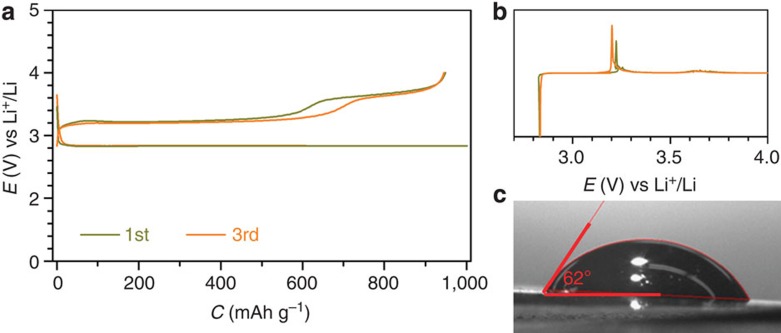
Initial three cycles of discharge and charge. (**a**) Discharge/charge profiles of the Li–O_2_ cells with a configuration of (Ru/MnO_2_/SP)/electrolyte/LiFePO_4_. The electrolyte is 0.5 M LiClO_4_ in DMSO with 120 p.p.m. H_2_O. (**b**,**c**) The corresponding d*Q*/d*V* curves and the contact angle of the electrolyte on the cathode. The discharge and charge cutoffs are 1,000 mAh g^−1^ (5,099 μC cm^−2^ based on the BET surface area of Ru/MnO_2_/SP) and 4.0 V, respectively. The potentials against Li^+^/Li are converted from LiFePO_4_. Rate: 500 mA g^−1^—based on the total weight of Ru, MnO_2_ and SP, corresponding to 0.71 μA cm^−2^; loading: ∼0.5 mg cm^−2^.

The carbon paper has been demonstrated to have negligible contribution to cell performance (Supplementary Fig. 1; Supplementary Note 1). The Li–O_2_ cell with Ru/MnO_2_/SP as cathode and the DMSO-based electrolyte containing p.p.m.-leveled H_2_O is discharged and charged at 500 mA g^−1^ (corresponding to 0.71 μA cm^−2^ based on the Brunauer-Emmett-Teller, (BET) surface area of Ru/MnO_2_/SP) as shown in Fig. 1a. A charge potential plateau as low as ∼3.20 V, corresponding to an overpotential of ∼0.24 V, can be clearly observed. Its corresponding d*Q*/d*V* curve in Fig. 1b shows sharp oxygen reduction reaction and oxygen evolution reaction peaks in the discharging and charging process, respectively, which are consistent with the flat discharge and charge plateaus. In addition, a small plateau at ∼3.60 V is also obtained in the charge profile in Fig. 1a. It could be resulted from the affinity of DMSO with the Ru/MnO_2_/SP cathode that has a large contact angle of 62°, compared with the discharge and charge profiles of the Li–O_2_ cell with a dimethoxyethane-(DME-)-based electrolyte and the good wettability of DME on the cathode in Supplementary Fig. 2. This may enable the oxygen evolved in a charging process to accumulate in the voids between the residual discharge product and the cathode, as schematically described in Supplementary Fig. 3 and Supplementary Note 2. It would induce slow diffusion of electrolytes into the voids for the decomposition of the remaining discharge products and result in the relatively large overpotential at ∼3.60 V, which is not observed in the affinitive DME-based electrolyte (Supplementary Fig. 2). Similar observations are also obtained in the other kinds of electrolytes, such as triglyme (G3)- and tetraglyme (G4)-based electrolytes (Supplementary Fig. 4). The trace amount of H_2_O in electrolytes is demonstrated to play a crucial role on reducing charge overpotentials of Li–O_2_ cells in all the widely used electrolytes, which was empirically considered as a negative factor in a Li-ion cell^29^.

### Analysis on the discharged/charged cathode

**Figure 2:**
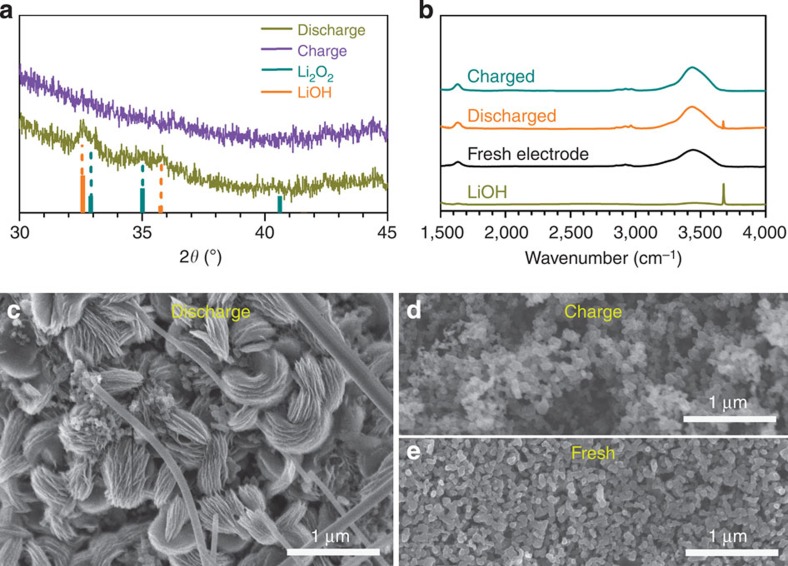
Characterization of the discharged/charged cathodes. (**a**) *Ex situ* XRD patterns of the discharged and charged Ru/MnO_2_/SP cathodes in DMSO-based electrolyte with 120 p.p.m. H_2_O. (**b**) IR spectra of the charged and discharge cathodes. (**c**,**d**) SEM images of the discharged and charged cathodes, in comparison to the fresh cathode (**e**).

The discharged and charged Ru/MnO_2_/SP cathodes in the DMSO-based electrolyte containing water have been characterized by X-ray diffraction (XRD). As shown in Fig. 2a, the discharge products are identified as a mixture of LiOH and Li_2_O_2_, referring to the standard powder diffraction files of 01-085-0777 and 00-009-0355, respectively. The diffraction peaks of LiOH become sharper and stronger at the cathode with a discharge capacity of 4,000 mAh g^−1^ and addition of more electrolyte (Supplementary Fig. 5), suggesting more LiOH converted from Li_2_O_2_. To avoid the effect of MnO_2_, quantification of LiOH and Li_2_O_2_ was conducted on the Ru/SP cathode with the same discharge capacity as in Fig. 1 via iodometric titration. The discharge product Li_2_O_2_ reacts with H_2_O via Li_2_O_2(s)_+2H_2_O_(l)_→H_2_O_2(l)_+2LiOH_(aq)_^30^,^31^, where H_2_O_2_ further oxidizes iodide to iodine, the titrated target. The LiOH and Li_2_O_2_ in the discharged cathode were titrated via two steps (Supplementary Figs 6 and 7; titration processes in Supplementary Methods) and estimated to 16.02 and 1.48 μmol, respectively, which are in agreement with the XRD patterns of the discharged cathode in Fig. 2a. The majority of LiOH formed at a discharged cathode is revealed by the characteristic absorbance peak in the infrared (IR) spectra in Fig. 2b. Based on the electrons passing through the cathode and the oxygen derived from the discharge products of LiOH and Li_2_O_2_ by iodometric titration, the discharging process is a 1.97 e^−^/O_2_ process, quite close to the theoretical value of 2.00 e^−^/O_2_. It is confirmed that in other electrolytes, like DME-, G3- and G4-based electrolytes, the discharging process is also a ∼2.00 e^−^/O_2_ process, as listed in Supplementary Table 1. This suggests that LiOH is converted from Li_2_O_2_ via a chemical, not an electrochemical, process in the discharging processes.

After charge, the discharge products LiOH and Li_2_O_2_ were decomposed, as evidenced by the disappearance of their characteristic diffraction peaks in Fig. 2a. Further, the discharge products were directly observed by scanning electron microscope (SEM) in Fig. 2c. All the products are toroidal particles with obvious layering. These are quite similar to the toroidal aggregates in a Li_2_O_2_-only discharged cathode^32^,^33^, which may suggest that the LiOH was derived from Li_2_O_2_ in the presence of H_2_O in electrolytes by persisting the similar shape. The charged cathode becomes more porous after decomposition of the discharge products in Fig. 2d in comparison to the fresh cathode in Fig. 2e. These results indicate that the low charge overpotentials demonstrated in Fig. 1 and Supplementary Fig. 3 resulted from the electrochemical decomposition of LiOH.

### Roles of Ru and MnO_2_ during discharge and charge

**Figure 3:**
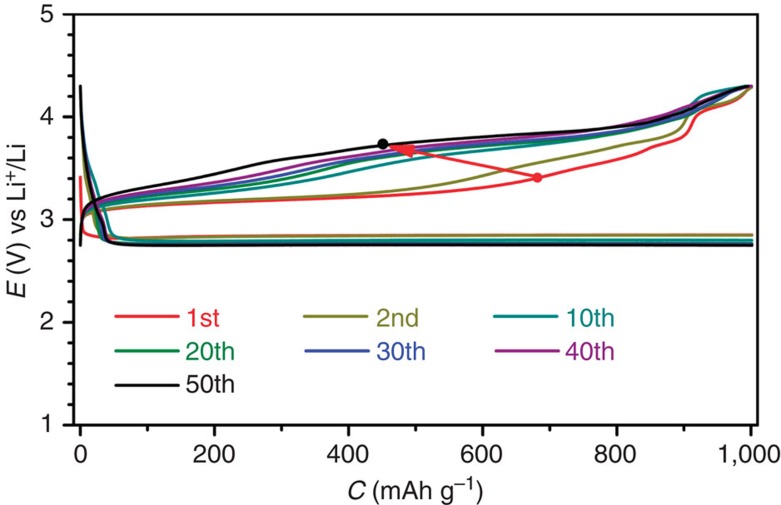
Discharge/charge profiles of the Li–O_2_ cell with Ru/SP. The charge potentials are steeply increased with cycles. Rate: 250 mA g^−1^.

In the Ru/MnO_2_/SP cathode, all the components of Ru^12^,^13^, MnO_2_ (ref. 34) and SP^35^ nanoparticles can act as oxygen reduction reaction catalysts. However, in the charging processes either SP or MnO_2_ supported on SP (MnO_2_/SP) did not show any plateaus at ∼3.2 V but instead at >3.6 V (Supplementary Figs 8 and 9). Only the Ru/SP as revealed in Supplementary Fig. 10 presented low charge potentials. This suggests good catalytic activity of Ru nanoparticles on the decomposition of LiOH. Fig. 3 shows 50 cycles of discharge and charge of the Li–O_2_ cell with the Ru/SP cathode. The charge potentials are sustained at ∼3.20 V in the initial few cycles, and then quickly are increased to ∼3.65 V in the tenth cycle and beyond. It indicates that the majority of the discharge product Li_2_O_2_ after multiple cycles cannot be converted to LiOH, because the p.p.m.-leveled H_2_O in the electrolyte was gradually consumed in the charging processes to produce H_2_O_2_. Hence, MnO_2_ (EMD), the best disproportionation catalyst of H_2_O_2_ (ref. 36), was incorporated into Ru/SP to regenerate H_2_O via 2H_2_O_2(l)_→2H_2_O_(l)_+O_2(g)_ and make H_2_O circulate during cycles and improve cycling stability of the Li–O_2_ cell.

### Rate capability and cycling stability

**Figure 4:**
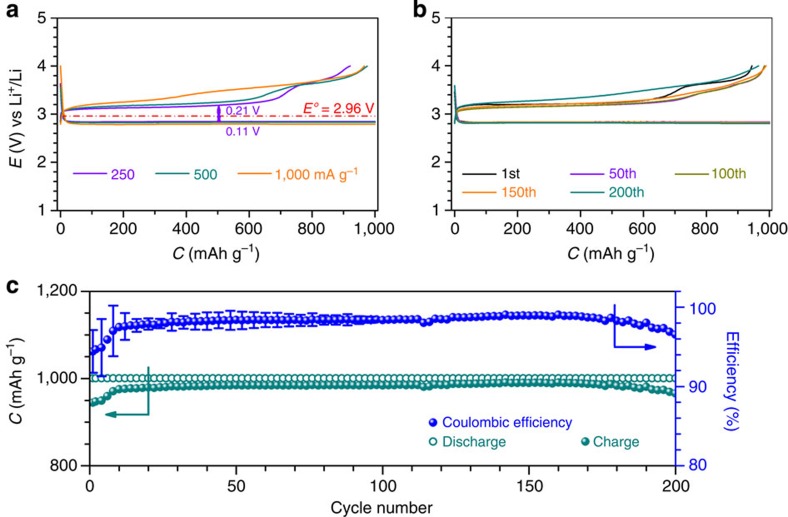
Rate capability and cycling performance of the Li–O_2_ cells with Ru/MnO_2_/SP. (**a**) Discharge/charge profiles of the tenth run at varied current densities from 250 to 500 and 1,000 mA g^−1^. (**b**) Discharge/charge profiles of the selected runs over the 200 cycles at 500 mA g^−1^. The cell was at rest for 1 min between each run. (**c**) Plot of discharge/charge capacities and the corresponding coulombic efficiencies against cycle number and error bars (s.e.m.) in the first 100 cycles.

The Li–O_2_ cell with Ru/MnO_2_/SP and the DMSO-based electrolyte containing p.p.m.-leveled H_2_O was examined at varied current densities, as shown in Fig. 4a. The polarization is obviously increased with the current density from 250 to 500 and 1,000 mA g^−1^. The overpotential in the discharging and charging process at 250 mA g^−1^ is 0.11 V and 0.21 V, respectively, leading to a small discharge/charge potential gap of 0.32 V. The Li–O_2_ cell was continuously discharged and charged at 500 mA g^−1^ for 200 cycles and ∼800 h, and the selected runs of discharge and charge are shown in Fig. 4b. The discharge and charge curves during the first 150 cycles are almost overlapped except the first charge. A slight charge potential increase beyond the 150 cycles can also be observed, which could be related to the electrolyte instability during many cycles (Supplementary Fig. 11)^37^,^38^. This indicates superior cycling stability of the Li–O_2_ cell, which is in sharp contrast to that without MnO_2_ in Fig. 3. The discharge and charge capacities in the 200 cycles are almost constant, and the corresponding coulombic efficiency in each run is approaching 98% (Fig. 4c), indicative of good reversibility. This may be partially benefited from the conversion of chemically active Li_2_O_2_ to LiOH. The reversible formation and decomposition of LiOH and Li_2_O_2_ during the 200 cycles are further confirmed by *ex situ* XRD patterns in Supplementary Fig. 12 and SEM images in Supplementary Fig. 13 (Supplementary Note 3). The low charge potentials sustained for so many cycles, to the best of our knowledge, have never been achieved before. The small discharge/charge potential gap and good cycling stability of the Li–O_2_ cell are rewarded by the ‘water catalysis' at the Ru/MnO_2_/SP cathode.

### Reaction mechanism

The equilibrium potential of LiOH in an aqueous solution is estimated to be ∼3.42 V vs Li^+^/Li based on Nernst equation^34^,^39^. However, in this studied electrolyte system the trace amount of H_2_O and LiClO_4_ are both the solutes and DMSO is the solvent. The equilibrium potential of LiOH is dependent on the concentration of H_2_O, and it can be roughly estimated to be ∼3.20 V, considering a concentration of 100 p.p.m. of H_2_O in the electrolyte (see the estimation process in Supplementary Methods). This is in good agreement with the observed low charge potential plateaus in all the widely employed electrolytes DMSO-, DME-, G3- and G4-based electrolytes for Li–O_2_ cells in Figs 1 and 4 and Supplementary Fig. 3.

**Figure 5:**
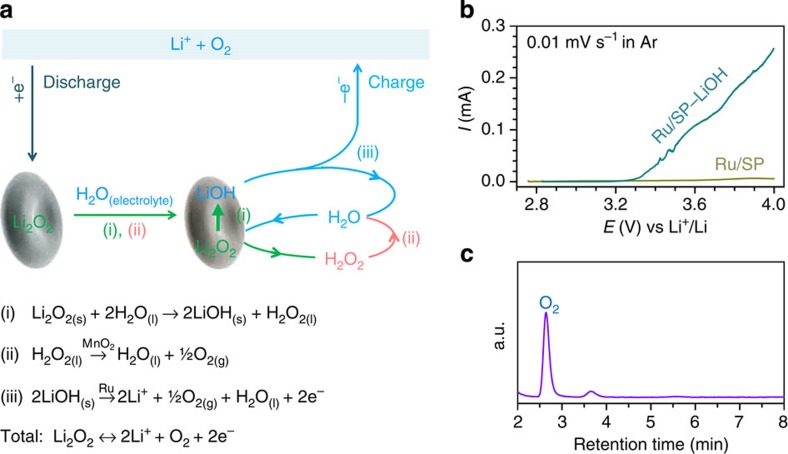
Proposed reaction mechanism, LSV and gas analysis. (**a**) (i) is a spontaneous process; (ii) is promoted over MnO_2_ nanoparticles in Ru/MnO_2_/SP; and oxidation of LiOH in (iii) occurs at low charge overpotentials over Ru nanoparticles. (**b**) Linear scanning voltammetry (LSV) curves of the Ru/SP electrodes with and without LiOH under Ar atmosphere. (**c**) Gas chromatography (GC) analysis on the gas evolved in a charging process.

Based on the above results, a mechanism for the discharging and charging process of the cell with Ru/MnO_2_/SP and the electrolyte containing a trace amount of H_2_O can be proposed and schematically described in Fig. 5a. On discharging, O_2_ accepts electrons via the external circuit and is reduced to generate the primary discharge product Li_2_O_2_ (refs 40, 41, 42). At the same time, the Li_2_O_2_ reacts with H_2_O from the electrolyte and is converted to LiOH via Steps (i and ii). Although Step (i) is an equilibrium^26^, it can be largely promoted to move forward by the conversion of one of the products H_2_O_2_ to H_2_O over MnO_2_ via Step (ii)^36^. The two Steps (i and ii) occur sequentially, and quickly transform Li_2_O_2_ to LiOH as long as H_2_O remains in the electrolyte. This has been confirmed by the presence of substantial LiOH in discharged cathodes as revealed by both of the XRD patterns and the IR spectra in Fig. 2. This is consistent with the observations of Aetukuri *et al.*^25^, and Schwenke *et al.*^26^, where the H_2_O in electrolytes is possibly consumed by the employed Li anode or saturated by the product LiOH, and the lack of a promoter like MnO_2_ for Step (ii) made the equilibrium reaction in Step (i) to move backward and result in the major discharge product Li_2_O_2_ as detected.

In the following charging process, the resultant LiOH can be directly oxidized via Step (iii) to regenerate H_2_O at low charge potentials, by which the residual Li_2_O_2_ is then converted to LiOH via Steps (i and ii) and oxidized. To demonstrate the feasibility of Step (iii), commercial LiOH was ball-milled and then thoroughly mixed with Ru/SP with a ratio of 30:70 (wt/wt). The electrodes of Ru/SP with and without LiOH under Ar atmosphere were subjected to linear scanning voltammetry (LSV) at a low scan rate of 0.01 mV s^−1^ as depicted in Fig. 5b. The Ru/SP electrode incorporated with LiOH presents significant oxidation currents and an oxidation onset potential of ∼3.27 V in Fig. 5b, which is in sharp contrast with no oxidation response on the electrode without LiOH. At a carbon electrode pre-filled with LiOH, a high charge potential of >4.0 V was reported^43^. This suggests that over Ru/SP LiOH was oxidized at low potentials, though it may be dependent on its morphology and facet orientation and the catalyst. The gas evolved in a charging process can be identified as O_2_ by a gas chromatography (GC) mounted with a thermal conductivity detector, as evidenced by the sharp GC signal of O_2_ in Fig. 5c. As shown in the whole discharging and charging process in Fig. 5a, H_2_O is not consumed and can be circulated to behave like a catalyst. The total electrochemical reaction occurring at the oxygen cathode is 2Li^+^+O_2_+2e^−^↔Li_2_O_2_, consistent with the Li–O_2_ cell chemistry.

## Discussion

**Figure 6:**
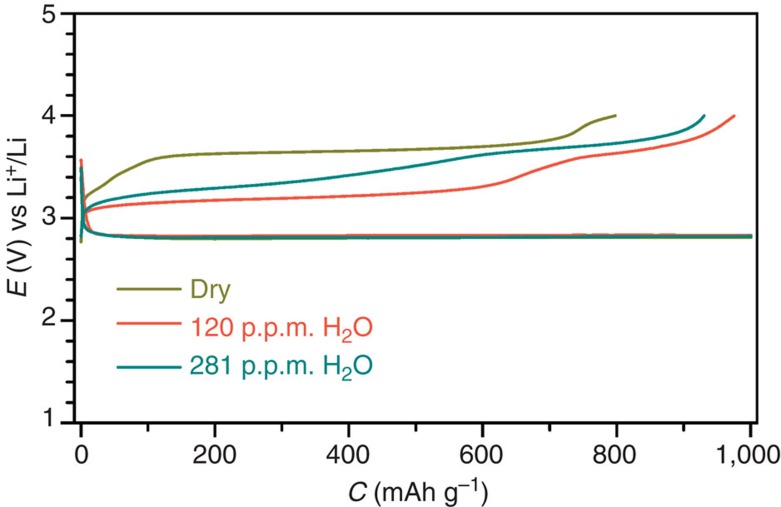
Discharge/charge profiles of the fifth cycles of the Li–O_2_ cells with Ru/MnO_2_/SP. The applied DMSO-based electrolytes are dried over a Li foil, and contain 120 and 281 p.p.m. of H_2_O. Rate: 500 mA g^−1^.

The dependence of the charge potentials on the concentration of H_2_O in electrolytes is shown in Fig. 6. In the dried DMSO-based electrolyte, one charge potential plateau can be obtained at ∼3.65 V, which is attributed to the oxidation of Li_2_O_2_ and in good agreement with the literatrues^10^,^11^,^12^,^13^,^14^,^15^. When there is 120 p.p.m. of water in the electrolyte, the charge potential plateau is significantly reduced to ∼3.2 V. This has been confirmed to the oxidation of LiOH that can be quickly converted from the primary discharge product Li_2_O_2_ via the sequential Steps (i and ii) in Fig. 5a over the catalyst of Ru/MnO_2_/SP in both the discharging and charging processes. However, when the concentration of water in the electrolyte is increased to 281 p.p.m., the charge potential plateau is shortened and increased. It may be induced by the LiOH on the surface of the discharge product, which is surrounded/adsorbed by H_2_O molecules in the electrolyte, and hence has high oxidation potentials following the Nernst equation^17^,^32^,^33^.

**Figure 7:**
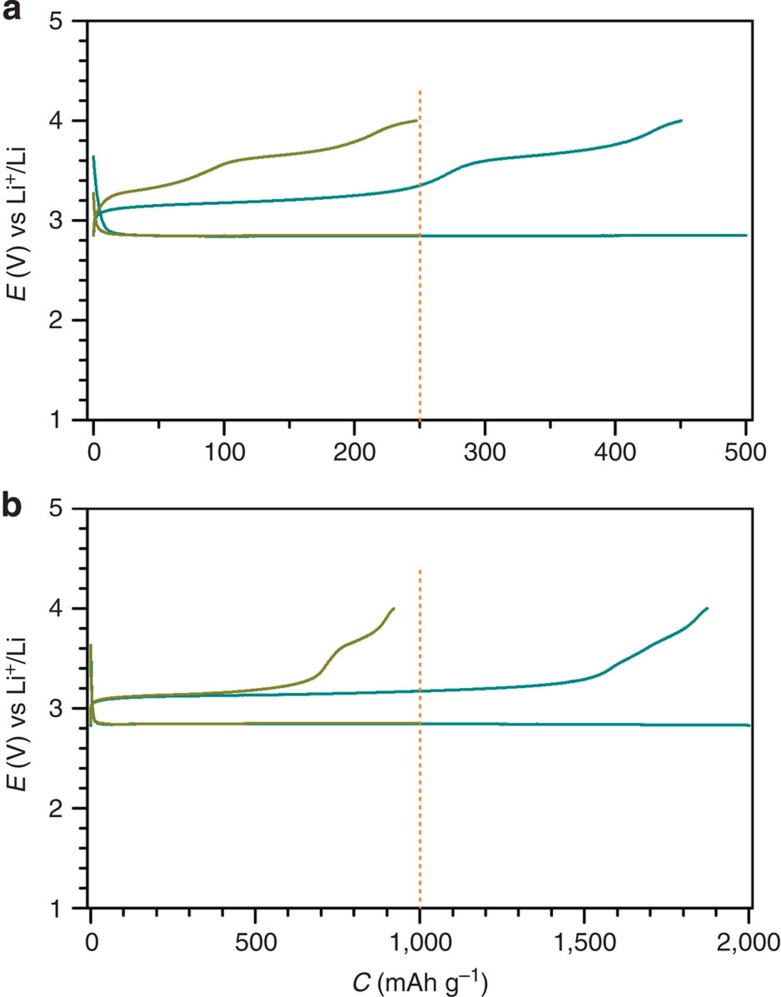
Discharge/charge profiles of the fifth cycles of the Li–O_2_ cell at 250 mA g^−1^. (**a**) Discharge capacity is limited for 250 and 500 mAh g^−1^. (**b**) Discharge capacity is limited for 1,000 and 2,000 mAh g^−1^. The cathode is Ru/MnO_2_/SP and the DMSO-based electrolyte containing 120 p.p.m. of H_2_O is applied.

In addition, by controlling the discharge depth of the Li–O_2_ cells, the discharge product can be ranged from LiOH only to a majority of Li_2_O_2_ plus LiOH with the same amount of electrolytes used in cells. The cathode with a relatively small discharge capacity of 250 mAh g^−1^ was covered with LiOH, on which no Li_2_O_2_ was detected by iodometric titration. It is in the shape of thin disks as shown in Supplementary Fig. 14 (refs 25, 33). In the following charging process, the potentials are increased to ∼3.65 V and similar to that with 281 p.p.m. of H_2_O in electrolytes in Fig. 6. The low charge potentials at ∼3.20 V can be observed again when the discharge capacity is increased to 500, 1,000 and 2,000 mAh g^−1^, as shown in Fig. 7. At large discharge capacities, the H_2_O molecules in the electrolyte can be consumed by Li_2_O_2_, producing surface-clean LiOH/Li_2_O_2_ in the cathode. This suggests that the relative amounts of H_2_O, LiOH and Li_2_O_2_ coexisting at a cathode side affect the charging overpotentials. The detailed formation/decomposition mechanism of LiOH and Li_2_O_2_ in the presence of water in electrolytes and the dependence of their decomposition potentials on their morphologies deserve further investigation.

The ‘water catalysis' at the oxygen cathode side has been demonstrated to reduce the charge overpotentials to ∼0.24 V, corresponding to ∼3.20 V vs Li^+^/Li and provides a possible solution to the current challenges of Li–O_2_ cells. With the Ru/MnO_2_/SP cathode and the DMSO-based electrolyte containing p.p.m.-leveled H_2_O, the Li–O_2_ cell presents a small discharge/charge potential gap of 0.32 V and superior cycling stability of 200 cycles ∼800 h. These have not been achieved before, and are rewarded by the proposed reaction mechanism. Although the LiFePO_4_ applied in cells is not a practical anode of Li–O_2_ cells, the reaction mechanism at the oxygen cathode may be extended to a practical Li–O_2_ cell by using a Li^+^ ion-conducting ceramic membrane to separate the electrolyte with water and the Li anode^2^,^39^. This could also alleviate the carbon-related side reactions by converting the chemically active Li_2_O_2_ to LiOH, and it will make the cheap and lightweight carbon possible as cathodes in Li–O_2_ cells, which has been suggested to avoid. This investigation will enable the cell to operate in ambient air by eliminating CO_2_ and advance the Li–O_2_/air cell technology.

## Methods

### Materials preparation

Electrolytic MnO_2_ (EMD, γ-MnO_2_) was received from TOSOH, Japan and ball-milled to nanometre scale. Ru/SP was synthesized as follows: 85 mg of SP was stirred into a solution of ethylene glycol (EG, purity of >99.5%, Wako Chemicals) containing 7.5 mg of Ru in RuCl_3_·xH_2_O (purity of 36∼42 wt% based on Ru, Wako Chemicals), and its pH was adjusted to 13 with 0.1 M of NaOH in EG. The suspension was then heated to 160 °C for 3 h with flowing N_2_. After cooling to 80 °C, its pH was adjusted to 3 using 0.1 M HCl and the resulting mixture was further stirred for 12 h. The final product Ru/SP was centrifuged, washed with de-ionized water until the solution pH reached ∼7 and dried at 80 °C in a vacuum oven for 12 h. For the preparation of MnO_2_/Ru/SP, the as-synthesized Ru/SP and MnO_2_ nanoparticles with a weight ratio Ru/MnO_2_/SP=7.5:7.5:85 were sonicated into an ethanol aqueous solution for 3 h and stirred overnight. Then, the product MnO_2_/Ru/SP was collected and dried at 80 °C under vacuum for 12 h.

DMSO, DME, G3 and G4 were firstly dried over 4 Å molecular sieves and then using Li metal. The Li salts of LiClO_4_ and lithium bis(fluorosulfonyl)imide were used as received from Wako Chemicals. The water in the prepared electrolytes was from the Li salts and measured on a desk-top Karl–Fisher Titration instrument.

### Cell assembly

The electrode film composed of Ru/MnO_2_/SP or Ru/SP and PTFE (A dispersion of 60 wt%, Du Pont-Mitsui Fluorochemicals Co. Ltd.) with a ratio of 85:15 wt% was rolled with a glass rod. The mass loading of Ru/MnO_2_/SP or Ru/SP is ∼0.5 mg cm^−2^. The electrode film was pressed onto a hydrophobic carbon paper (SIGRACET Gas Diffusion Media, Type GDL 35BA) to work as a cathode. The anode was consisted of LiFePO_4_ (Sumitomo Osaka Cement), SP and PTFE (70/20/10) pressed on an Al mesh. The amount of DMSO- and DME-based electrolytes in a coin cell was 100 μl and G3- and G4-based electrolyte was 50 μl, considering their different vapour pressures. The employed separator was a glass microfibre filter paper (GF/A, Whatman). The Li–O_2_ cell assembly in 2032 coin cells was conducted in an Ar-filled glove box that has a dew point of around −90 °C (∼0.1 p.p.m. of H_2_O) and O_2_ content below 5 p.p.m.. The cells stored in a glass chamber with a volume capacity of 650 ml were purged with O_2_ (99.999%) before electrochemical tests.

### Characterization and measurements

The contact angles of electrolytes on the cathode were examined on AST Products with a model of Optima. The BET surface area of Ru/MnO_2_/SP was measured to be 70.6 m^2^ g^−1^ on Belsorp 18 via nitrogen adsorption–desorption. XRD was performed on a Bruker D8 Advanced diffractometer with Cu Kα (*λ*=1.5406 Å) radiation with a scan rate of 0.016° per s. Galvanostatic discharge/charge was conducted on a Hokuto discharging/charging system. All the electrochemical measurements were conducted at 25 °C. The specific capacities and currents are based on the mass of Ru/MnO_2_/SP or Ru/SP in cathodes. The discharged and charged cathodes were extracted in the glove box and washed with dried dimethoxyethane. SEM was obtained on Hitachi S4800. The Ru/MnO_2_/SP was mixed with KBr and then pressed to a pellet for Fourier transform infrared spectroscope (FTIR) characterization on a JASCO instrument of FT/IR-6,200 from 2,000 to 400 cm^−1^ with a resolution of 2 cm^−1^. The electrolytes during cycles were collected by washing the glass fibre filter separators with acetone-d_6_, and subjected to ^1^H NMR (Bruker, 500 MHz).

## Additional information

**How to cite this article:** Li, F. *et al.* The water catalysis at oxygen cathodes of lithium–oxygen cells. *Nat. Commun.* 6:7843 doi: 10.1038/ncomms8843 (2015).

## Supplementary Material

Supplementary Information Supplementary Figures 1-14, Supplementary Table 1, Supplementary Notes 1-3 and Supplementary Methods
