# Supplementary material for: The water catalysis at oxygen cathodes of lithium–oxygen cells
Source: Nat Commun. 2015 Jul 24;6:7843. doi: 10.1038/ncomms8843 (PMC4525193; doi:10.1038/ncomms8843)
Supplement: Supplementary Information — Supplementary Figures 1-14, Supplementary Table 1, Supplementary Notes 1-3 and Supplementary Methods [file ncomms8843-s1.pdf]

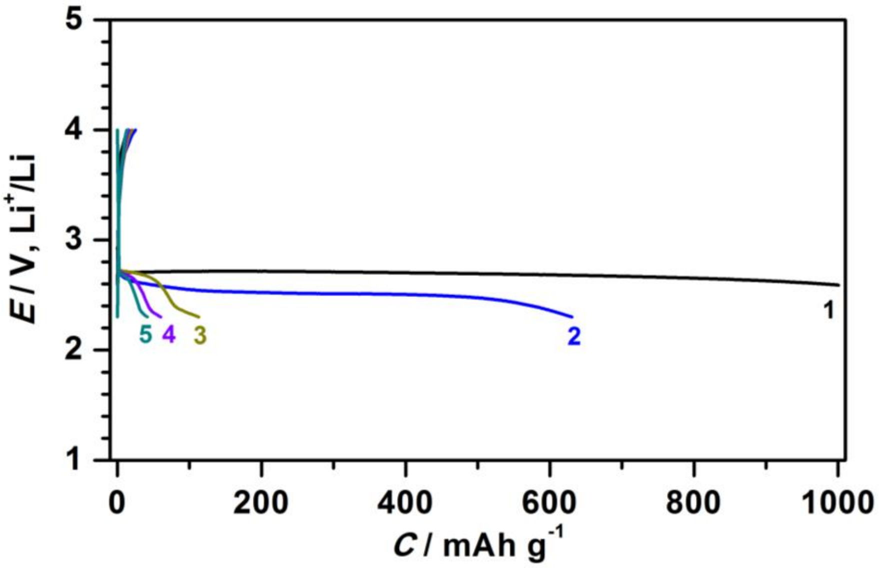

Supplementary Fig. 1 | Profiles of Discharge/charge cycles. The Li-O_2 cell has a configuration of carbon paper/electrolyte/$LiFePO_4$, and the employed electrolyte is the DMSO-based electrolyte containing 120 ppm of H_2O. The potentials against Li are converted from $LiFePO_4$. The applied current is 0.25 mA cm^{-2} based on the geometrical surface area of the carbon paper, which is corresponding to 500 mA g^{-1} when the Ru/MnO_2/SP loading is 0.5 mg cm^{-2}.

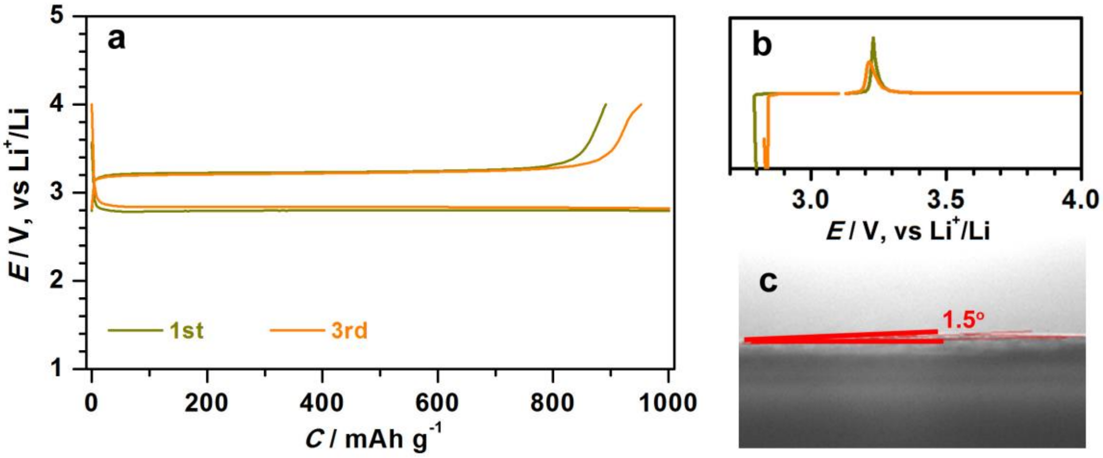

Supplementary Fig. 2 | Initial three cycles of discharge and charge. a, Discharge-charge profiles of the Li-O_2 cells in 0.1 M $LiClO_4$ in DME with 23 ppm H_2O. **b, c**, The corresponding dQ/dV curves and the contact angles of the electrolytes on the Ru/MnO_2/SP cathode. The discharge and charge cutoffs are 1000 mAh g^{-1} and 4.0 V, respectively. Rate: 500 mA g^{-1} based on the total weight of Ru, MnO_2 and SP; loading: ~ 0.5 mg cm^{-2}.

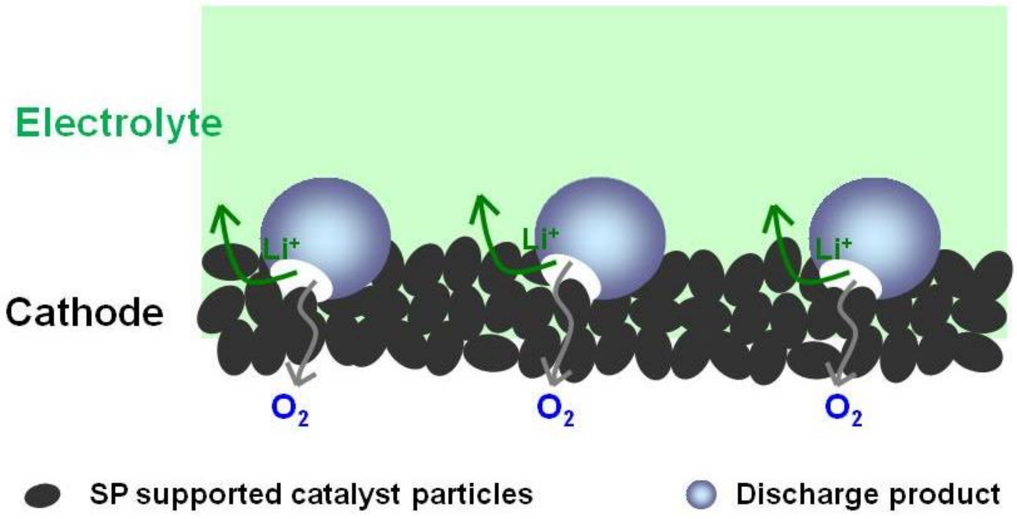

Supplementary Fig. 3 | Diffusion of Li^+ and O_2 at interfaces upon charging. The diffusion processes occur at the interfaces between electrolyte, discharge product Li_2O_2, and SP particles.

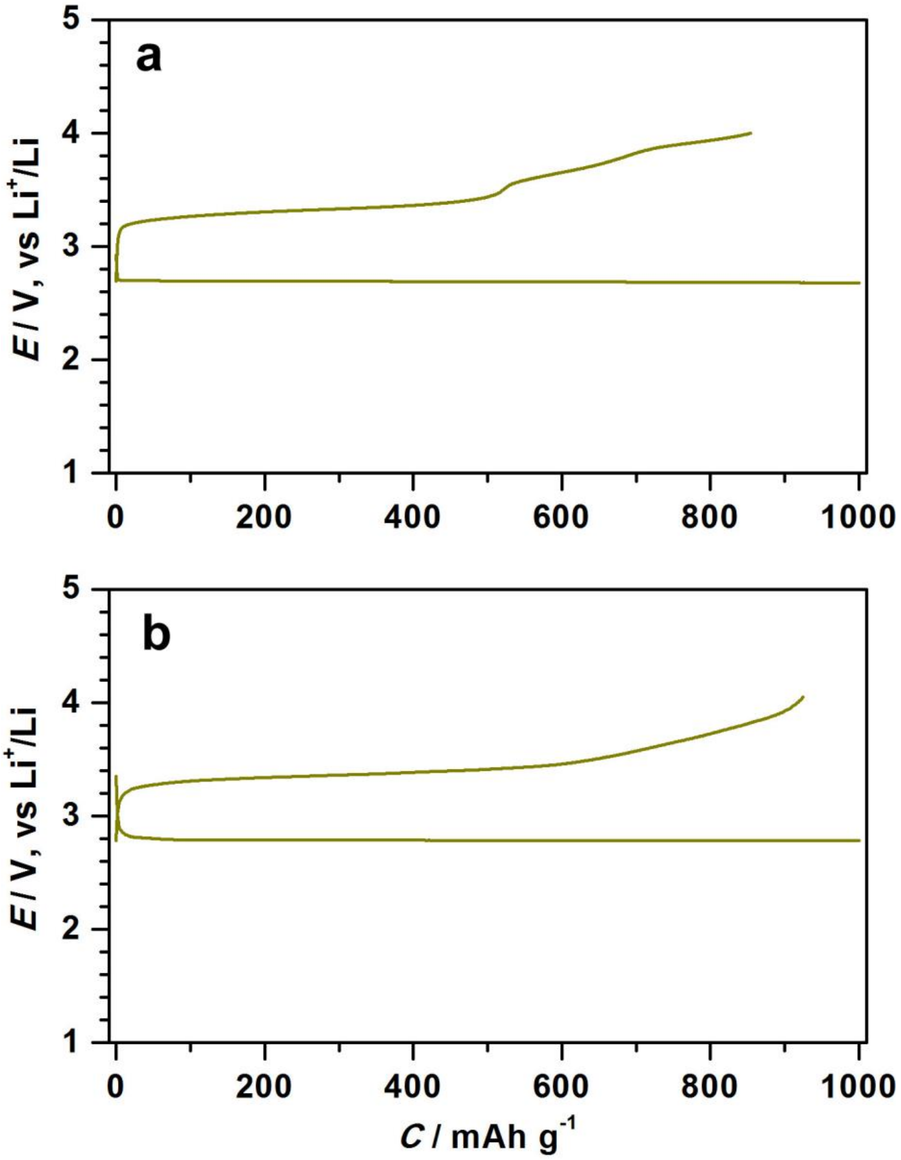

Supplementary Fig. 4 | Discharge and charge profiles. The Li-O_2 cells were discharged and charged at 250 mA g^{-1} in G3- (a) and G4- (b) based electrolytes containing 133 and 150 ppm of H_2O, respectively. The solubility of $LiClO_4$ in G3 is very limited, the Li salt is therefore changed to LiFSI in the G3-based electrolyte. The concentration of LiFSI in G3 is 0.5 mol L^{-1}.

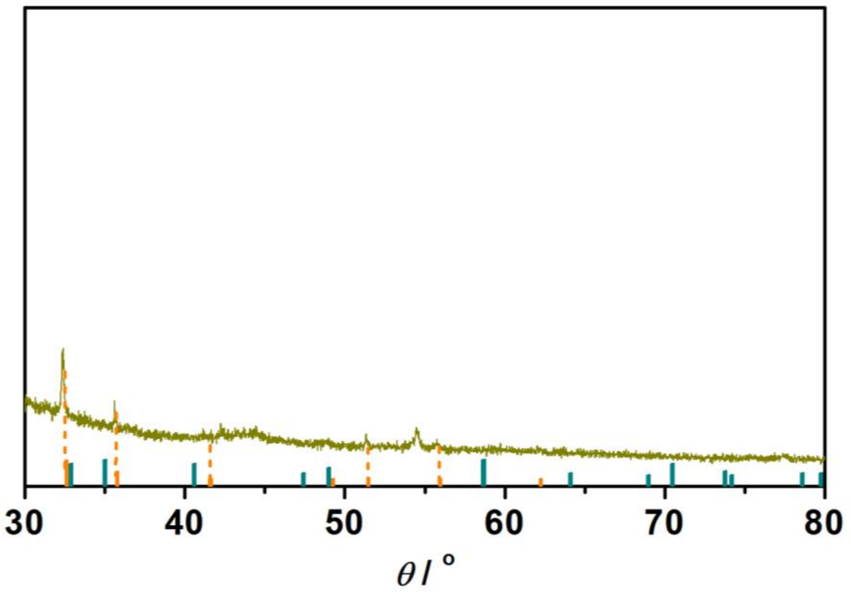

Supplementary Fig. 5 | *ex-situ* XRD pattern of the discharged Ru/MnO_2/SP cathode. 300 μL of the DMSO-based electrolyte with H_2O was used in the Li-O_2 cell, and the discharge capacity is 4000 mAh g^{-1}. The references are LiOH (red short dashed lines) and Li_2O_2 (dark cyan short dashed lines) with the standard powder diffraction files of 01-085-0777 and 00-009-0355, respectively. It indicates that LiOH is the major crystalline discharge product.

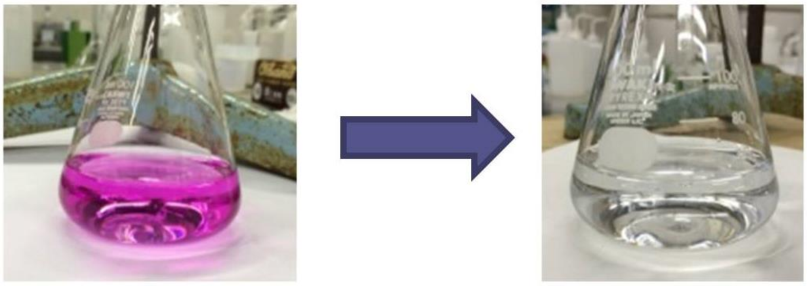

Supplementary Fig. 6 | Color changes during alkaline titration.

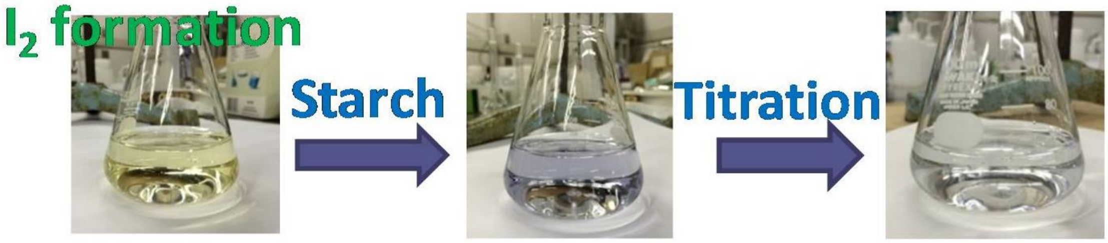

Supplementary Fig. 7 | Color changes during iodometric titration.

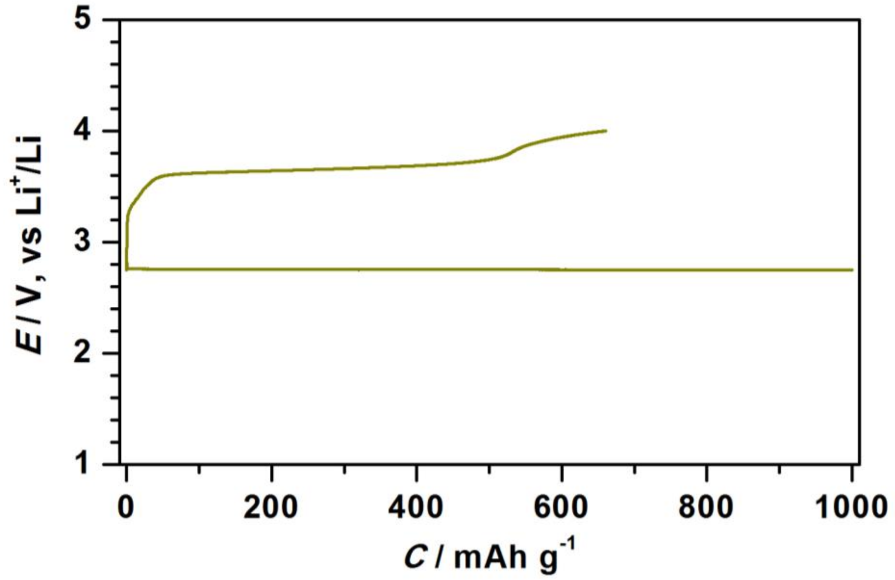

Supplementary Fig. 8 | Discharge-charge profile of the Li-O_2 cell with SP. The electrolyte is the DMSO-based electrolyte with H_2O. Rate: 100 mA g^{-1}.

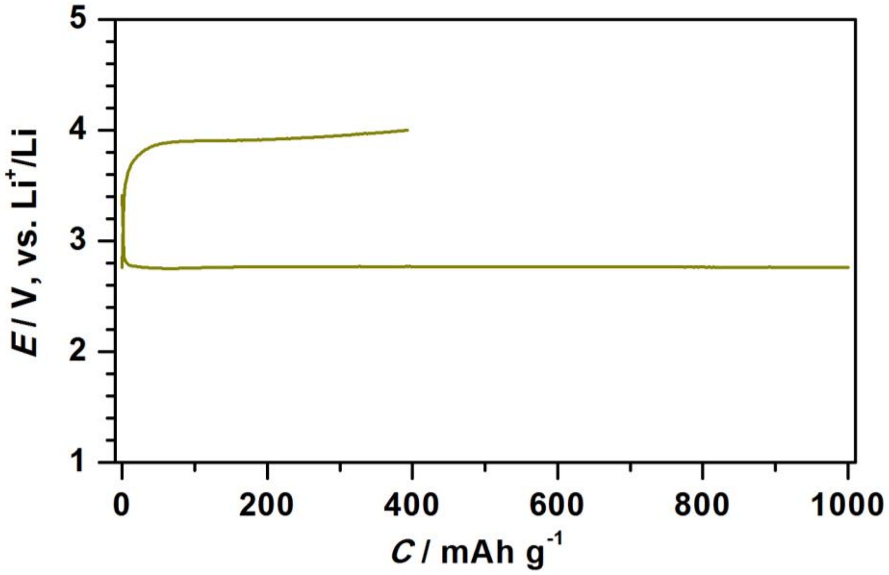

Supplementary Fig. 9 | Discharge-charge profile of the Li-O_2 cell with MnO_2/SP.

The electrolyte is the DMSO-based electrolyte with H_2O. Rate: 250 mA g^{-1}.

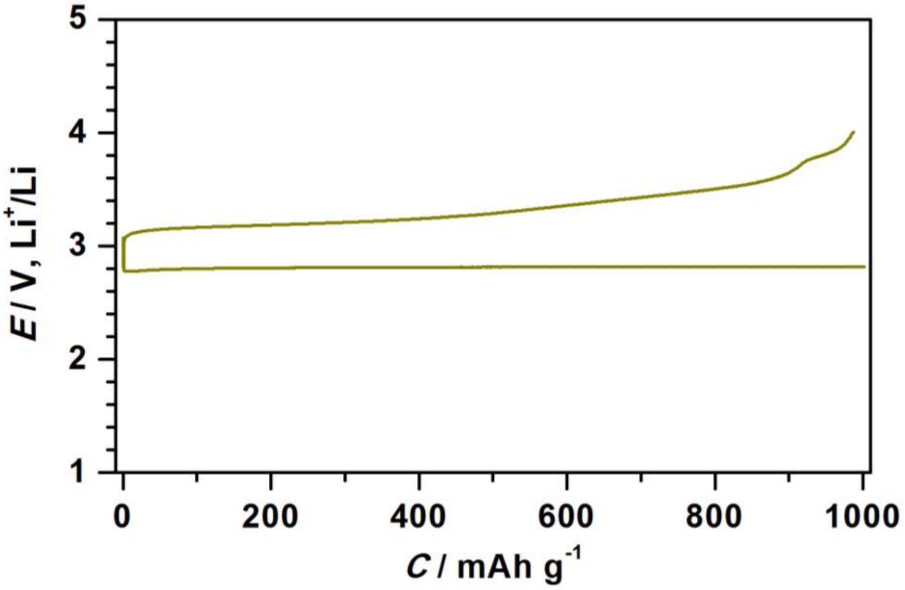

Supplementary Fig. 10 | Discharge-charge profile of the Li-O_2 cell with Ru/SP. The electrolyte is the DMSO-based electrolyte with H_2O. Rate: 250 mA g^{-1}.

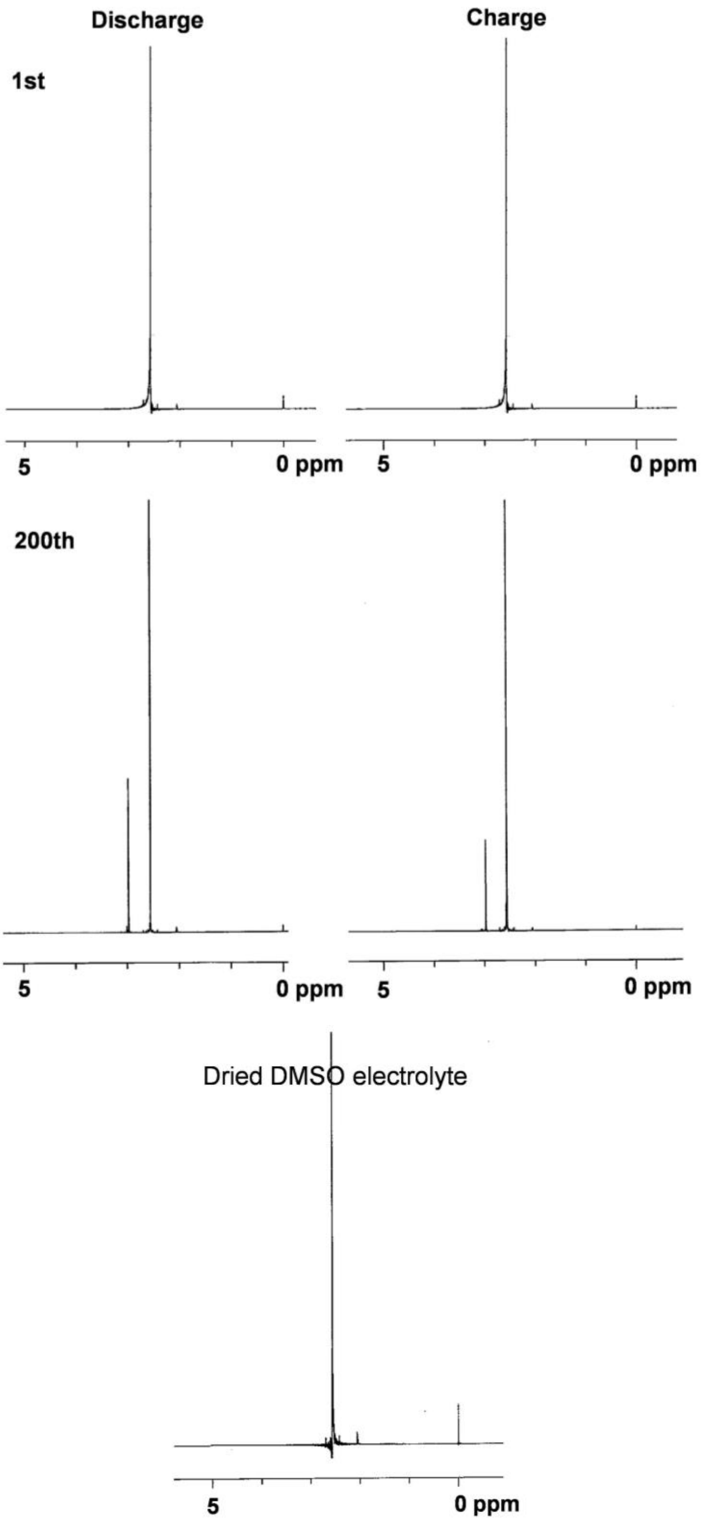

Supplementary Fig. 11 | ^{1}H NMR spectra. The electrolytes were extracted from the selected runs of discharge and charge, and the fresh DMSO electrolyte was dried over a Li foil. The main peak at ~ 2.6 ppm is ascribed to DMSO. The peak at ~ 3.0 ppm is corresponding to the byproduct dimethyl sulfone during cycles by comparing with its pure phase in the solvent acetone-d_6.

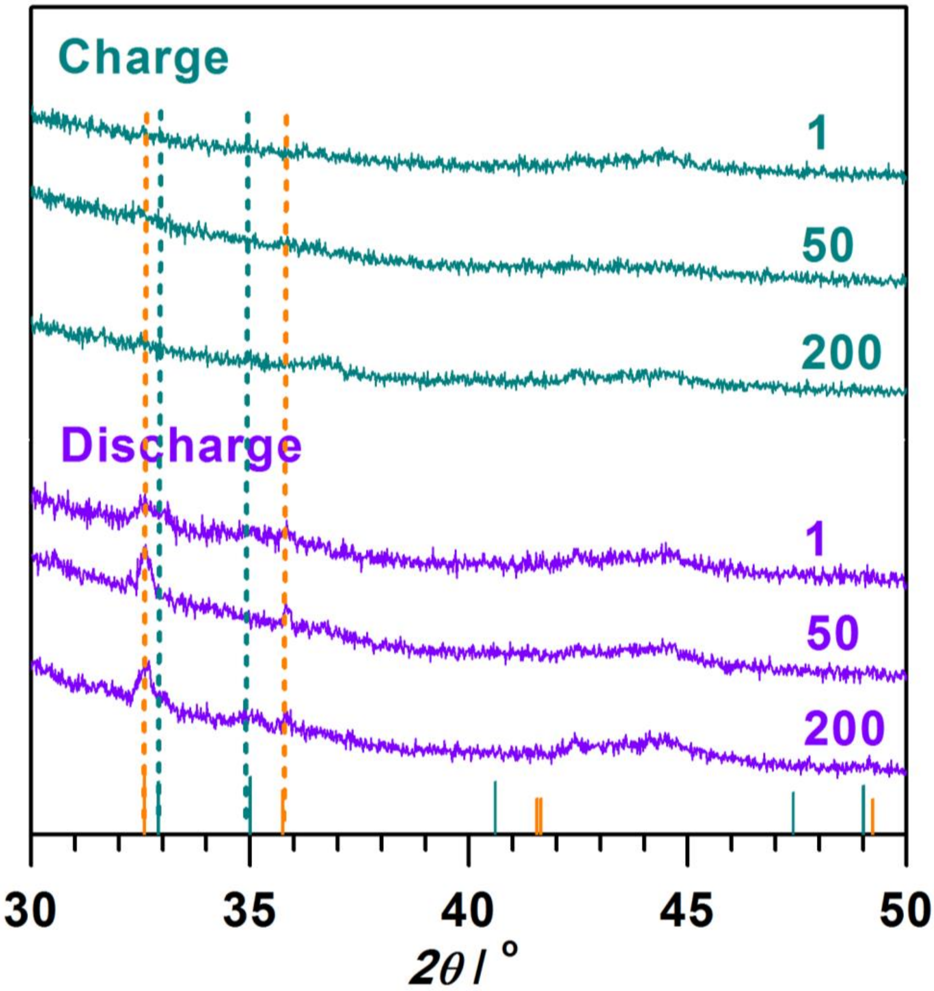

Supplementary Fig. 12 | *ex-situ* XRD patterns during cycles of discharge and charge. The discharge capacity of the cathode Ru/MnO_2/SP is limited at 1000 mAh g^{-1} and the charging voltage is set at 4.0 V, which are the same as those in Figure 4. The applied electrolyte is the DMSO-based electrolyte. The references are LiOH (red short dashed lines) and Li_2O_2 (dark cyan short dashed lines) with the standard powder diffraction files of 01-085-0777 and 00-009-0355, respectively.

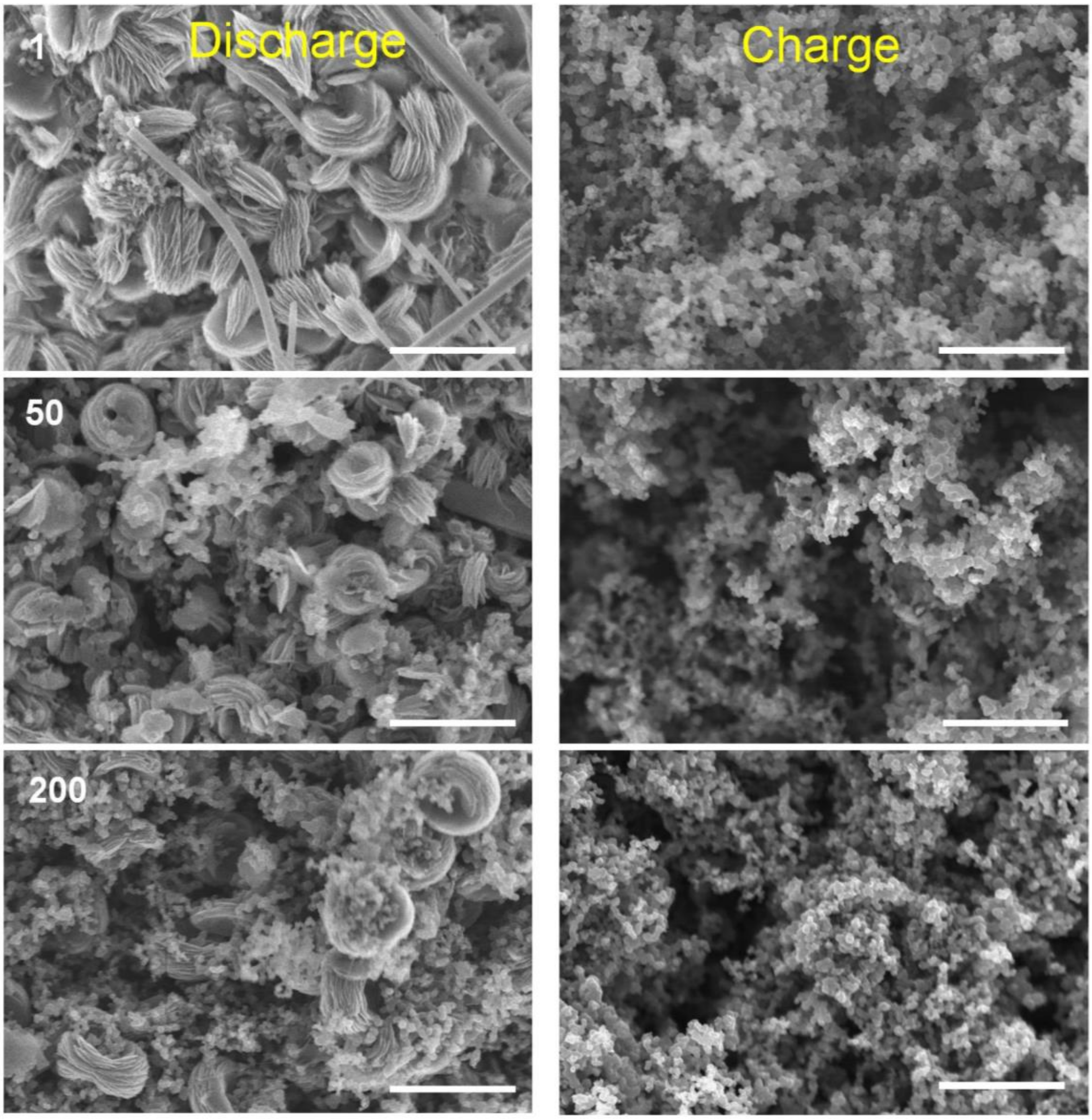

Supplementary Fig. 13 | SEM images of the discharged and charged Ru/MnO_2/SP cathodes during cycles. The discharge capacity is limited at 1000 mAh g^{-1} and the charging voltage is set at 4.0 V, which are the same as those in Figure 4. The applied electrolyte is the DMSO-based electrolyte. The scale bar is 1μm.

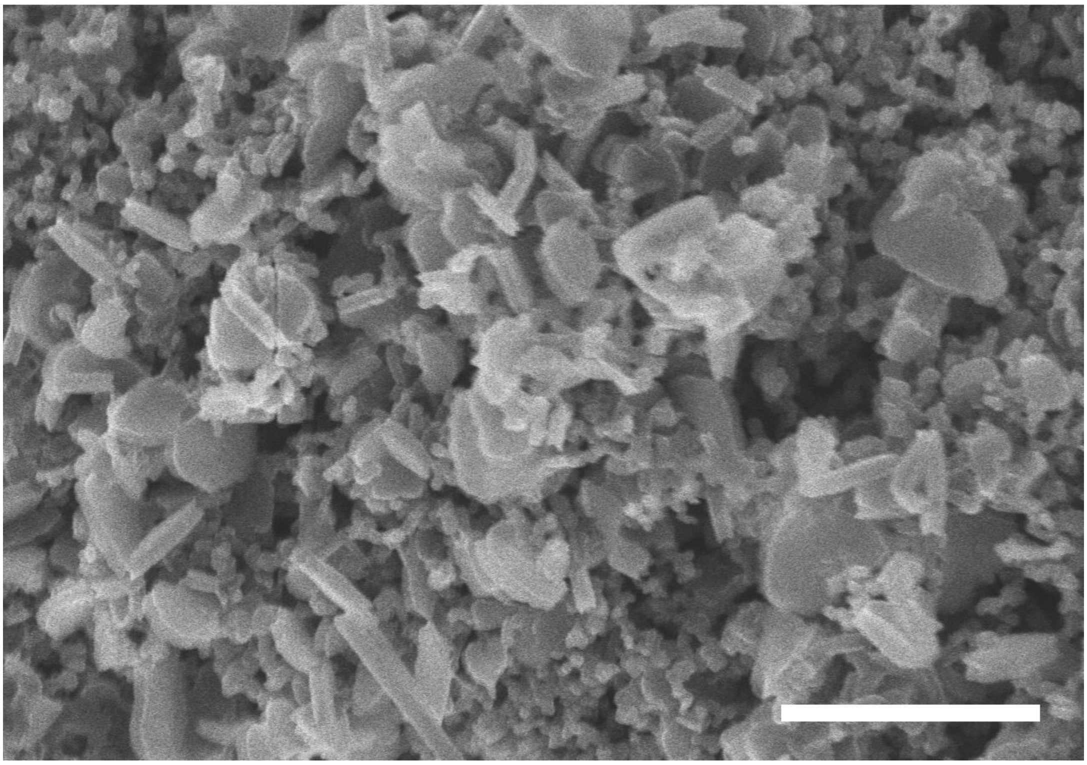

Supplementary Fig. 14 | SEM image of a discharged Ru/MnO_2/SP cathode. The discharge capacity is 250 mAh g^{-1}, and the current density is 250 mA g^{-1}. The scale bar is 1μm.

Supplementary Table 1 | Quantification of LiOH and Li_2O_2. The titration is conducted on Ru/SP in different electrolytes in the discharging processes.

	DMSO	DME	G3	G4
LiOH (μmol)	16.02	17.42	18.14	12.94
Li_2O_2 (μmol)	1.48	0.49	0.28	2.48
e^-/O_2	1.97	2.03	2.00	2.09

Discharge capacity: 0.5 mAh cm^{-2}. The amount of O_2 is derived from LiOH and Li_2O_2.

Supplementary Note 1

In Supplementary Fig. 1, the discharge potential of carbon paper is lower than that with the catalyst Ru/MnO_2/SP, and it cannot be charged when the upper potential limit is set 4.0 V. The discharge capacity of the carbon paper decays quickly. In comparison with the discharge/charge potentials and cycling stability of the Li-O_2 cell with Ru/MnO_2/SP as the cathode, the contribution of carbon paper can be neglected.

Supplementary Note 2

In Supplementary Fig. 3, the Li^+ ions have to diffuse into the residual pores created by the decomposition of discharge product at the porous cathode side to enable the continuation of the charging process. The various affinities of electrolytes to the porous cathode will induce different polarizations, which can accordingly lead to different overpotentials.

Supplementary Note 3

In Supplementary Fig. 13, in the initial few cycles, the discharge products LiOH and Li_2O_2 are mostly formed on the top of cathode, while after multiple cycles they are mostly formed in the pores beneath the cathode surface facing the electrolyte. The fibers in the SEM images are the glass fibers from the separator used in the cells.

Supplementary Methods

Titration of LiOH and Li_2O_2 in a discharged Ru/SP cathode. The titration contains two steps: (i) titration of LiOH and (ii) titration of I_2:

(i) The Ru/SP cathode was extracted from a discharged coin cell in an Ar-filled glove box, washed with dried DME and dried under vacuum. The cathode was taken out of the glove box, and put into a conical flask with 20 ml of de-ionized water immediately. The flask was vigorously shaken to promote the complete reaction of Li_2O_2 with H_2O. The alkaline solution was titrated with a standard 5 mmol L^{-1} of HCl, and the end point was indicated by 0.1 mL of phenolphthalein in isopropanol, as shown in Supplementary Fig. 6.

Involved reaction:

$$Li_2O_{2(s)} + 2H_2O_{(l)} \rightarrow H_2O_{2(l)} + 2LiOH_{(aq)} \quad (1)$$

(ii) After the alkaline titration was finished, three reagents were added into the existing solution in sequence: 0.5 mL of 2 wt% KI in H_2O, 0.1 mL of 60 wt% H_2SO_4, and 25 µL of a molybdate based catalyst solution. The catalyst solution was prepared by dissolving 0.5 g ammonium molybdate in 5 mL of 30 wt% ammonia aqueous solution, followed by adding 1.5 g of ammonium nitrate and diluting the solution to 25 mL total using de-ionized water. The resultant solution turns yellow upon reagent addition due to I_2 formation, and the I_2 was immediately titrated to a pale yellow color using 1 mmol L^{-1} of $Na_2S_2O_3$ solution. At this point, 0.1 mL of 5 wt% starch indicator was added, which turned blue, and the titration was continued until the color disappeared. The color changes during the titration are shown in Supplementary Fig. 7. The amount of LiOH estimated from the titration was consistent with the trace amount of H_2O in the electrolyte.

Involved reactions:

$$H_2O_2 + 2KI + H_2SO_4 \rightarrow I_2 + K_2SO_4 + 2H_2O \quad (2)$$ over molybdate based catalyst

$I_2 + 2Na_2S_2O_3 \rightarrow Na_2S_4O_6 + 2NaI$ (3)

Rough estimation of the charge potentials. The reactions occurring at a cathode and an anode and the potentials's dependence are described below:

Total: $LiOH \rightarrow Li + ½H_2O + ¼O_2$

(+) $LiOH \rightarrow Li^+ + ½H_2O + ¼O_2 + e^-$

$\varphi_+ = \varphi_{O_2/LiOH}^o + 0.059\lg\alpha_{Li^+} + 0.059 \times ½\lg\alpha_{H_2O}$

(-) $Li^+ + e^- \rightarrow Li$

$\varphi_- = \varphi_{Li^+/Li}^o + 0.059\lg \alpha_{Li^+}$

Charge potential: $\varphi = \varphi_+ - \varphi_- = (\varphi_{O_2/LiOH}^o - \varphi_{Li^+/Li}^o) + 0.059 \times ½\lg\alpha_{H_2O}$

Standard potential estimation based on Gibbs free energy

$LiOH \longrightarrow Li + ¼O_2 + ½H_2O$

G^o -438.96 -237.14 kJ/mol

$E^o = -\Delta G^o/F = 3.32$ V

$E = E^o + 0.059 \times ½\lg\alpha_{H_2O}$

For example, $[H_2O]$ = **10 ~ 100 ppm**

$E \approx$ **3.17 ~ 3.20 V**

For example, in aqueous solution, $E \approx 3.32 + 0.059\lg55.6 \approx$ **3.42 V**

(α: activity)

In the estimation process, the concentration of O_2 dissolved in electrolyte is not considered. The initial concentration of H_2O in the electrolyte is used to estimate the equilibrium potential of LiOH in the charging process, without considering that H_2O has been completely or partially converted to LiOH in the discharging process.
